# More Than Maintaining Competence: A Qualitative Study of How Physicians Conceptualize and Engage in Lifelong Learning

**DOI:** 10.5334/pme.1327

**Published:** 2024-07-04

**Authors:** Bridget C. O’Brien, Sally Collins, Lindsey M. Haddock, Sara Sani, Josette A. Rivera

**Affiliations:** 1 Professor in the Department of Medicine and an education scientist, Center for Faculty Educators, University of California, San Francisco, San Francisco, California, US; 2 Research associate with the Center for Faculty Educators, University of California, San Francisco, California, US; 3 Clinical assistant professor in the Section of Geriatrics, Division of Primary Care and Population Health, Department of Medicine at Stanford University School of Medicine, Stanford, California, US; 4 Assistant clinical professor in the Divisions of Hospital and Emergency Medicine, Department of Medicine, San Francisco Veterans Affairs, San Francisco, California, US; 5 Professor in the Division of Geriatrics, Department of Medicine, University of California, San Francisco, California, US

## Abstract

**Purpose:**

Physicians have a professional responsibility to engage in lifelong learning. Some of this lifelong learning is required to maintain licensure and certification. Yet, this conceptualization captures only a small portion of the content areas and learning processes that physicians need to engage with to ensure quality patient care. Additionally, purposes beyond regulatory requirements and professional obligations likely drive physicians lifelong learning, though these purposes have not been explored. Given the centrality of lifelong learning to quality patient care, our study explores how physicians conceptualize and engage in lifelong learning.

**Method:**

We conducted a qualitative interview study using an interpretivist approach. In 2019, we recruited 34 academic physicians from one institution. We analyzed our data to identify themes related to conceptualization of purposes, content areas, and processes of lifelong learning and actual lifelong learning practices.

**Results:**

We interpreted participants’ descriptions and examples of lifelong learning as serving three purposes: maintaining competence, supporting personal growth and fulfillment, and engaging in professional stewardship. Much of participants’ discussion of lifelong learning centered around keeping up to date with medical knowledge and clinical/procedural skills, though some also mentioned efforts to improve communication, leadership, and teamwork. Participants engaged in lifelong learning through contextual, social, and individual processes.

**Discussion:**

Academic physicians engage in lifelong learning for reasons beyond maintaining competence. Medical knowledge and clinical/procedural skills receive most attention, though other areas are recognized as important. Our findings highlight opportunities for a broader, more comprehensive approach to lifelong learning that spans all areas of medical practice.

## Introduction

When formal education ends and trainees become licensed professionals, their learning must continue. This professional responsibility is widely recognized as lifelong learning [1,2], which ideally spans all domains of professional practice (e.g., medical knowledge, clinical skills, communication, professionalism) [3,4]. The content areas and processes physicians choose when engaging in lifelong learning matter not only for the current quality of medical practice, but also for the practices and standards instilled in future generations of physicians as they learn from role models and supervisors.

How physicians conceptualize lifelong learning plays an important role in their lifelong learning practices. This conceptualization includes content areas and processes as well as the purpose of lifelong learning. A narrow conceptualization of content might be limited to medical knowledge and clinical or procedural skills, while a broader conceptualization would encompass additional areas relevant to medical practice such as communication, professionalism, equity, and emotional awareness. Much of the literature on lifelong learning has examined a narrow range of content areas, perhaps because medical knowledge and clinical skills are tested on maintenance of certification examinations [5] and constitute most continuing medical education (CME) offerings [6,7]. These narrow conceptualizations are problematic because they limit where and how physicians focus their attention and effort, potentially missing opportunities to develop in areas of need beyond medical knowledge and clinical skills. Growing concern about patient dissatisfaction and distrust of physicians [8,9], biases and inequities in care [10,11,12], and physician wellbeing and burnout [13,14,15] underscores the importance of physicians’ engagement in lifelong learning patient-centered care, social and structural determinants of health, and well-being/self-care.

Lifelong learning processes can also be narrowly or broadly construed, with a narrower conceptualization focusing on formal learning processes such as trainings, courses, or didactics that are part of continuing medical education (CME) or continuing professional development (CPD) offerings and a broader conceptualization including informal learning processes such as reading articles, observing others, and talking with colleagues [16,17,18]. While the literature on lifelong learning in medicine is large, many studies examine formal settings in an effort to evaluate the effectiveness of CME and CPD offerings [19,20]. This narrow focus limits our understanding of lifelong learning processes in daily practice and further limits the content areas studied since most content covered in these formal offerings is medical knowledge and clinical or procedural skills. A few recent studies recommend recognizing informal learning processes (e.g, conversations with colleagues or reading recent literature) as part of physicians’ CPD [16,17,21,22] because they are accessible forms of lifelong learning in the workplace. A broader conceptualization that includes and recognizes the value of informal learning processes can better support the full range of opportunities for lifelong learning.

While prior literature gives us some insight into how physicians conceptualize the content and processes of lifelong learning, even if narrowly construed, the literature offers little insight into how physicians conceptualize the purpose, or why, they engage in lifelong learning.

While the medical profession tends to focus on maintaining competence as the primary purpose of lifelong learning [23], the broader literature on adult and continuing education describes multiple purposes of lifelong learning. Education researcher and philosopher Gert Biesta discusses three “agendas,” or purposes, of lifelong learning: a functional or economic purpose that is externally motivated and related to keeping up-to-date with the knowledge and skills needed for employment; a personal growth and fulfillment purpose that is intrinsically motivated and gives meaning and value to life; and a social or democratic purpose whereby lifelong learning enables contributions to social improvement and participation in democratic processes [24]. Medicine’s emphasis on maintaining competence, while clearly important, may undermine other valuable purposes physicians associate with lifelong learning – purposes that are crucial to their continued engagement and satisfaction at work.

Understanding how physicians conceptualize and engage in lifelong learning and to what extent their actual learning practices span a broad array of processes and content areas relevant to medical practice is important because their beliefs and actions are the foundation for the expectations and norms influencing trainees and may have consequences for quality patient care and physician well-being.

Our study aims to enrich understanding of lifelong learning by exploring:

1. how physicians conceptualize the purpose, content, and processes of lifelong learning.
2. how physicians engage in lifelong learning, particularly for aspects of practice other than medical knowledge and clinical/procedural skills.

## Methods

We conducted a qualitative study using an interpretivist approach [25]. Interpretivist approaches view knowledge as a product of meaning-making through social interaction and reality as subject to multiple interpretations. As such, we recognize that the perspectives and experiences we bring to the research shape the information collected and our interpretations of data. Our interdisciplinary team consists of three physicians with first-hand experience of lifelong learning in clinical practice (LH, SS, JR), a research associate in health professions education with clinical experience as a speech pathologist (SC), and a researcher in health professions education with no clinical experience (BO’B). Our different perspectives occasionally led to different interpretations of participants’ words, which prompted further discussion and more nuanced understanding of meaning that helped us move from a descriptive coding to thematic synthesis.

### Theoretical orientation

The primary theories guiding most research on lifelong learning include self-directed or self-regulated learning [26], deliberate practice [27], and workplace learning [28]. Self-directed, self-regulated learning and deliberate practice tend to focus on cognitive processes and align well with competency-based perspectives. They begin with an ideal model of learning and compare what physicians do with this model. Workplace learning theories are practice-oriented, rooted in sociocultural and social learning perspectives, exploring the context and beliefs that guide what people do in practice and offering insights into their lifelong learning processes. Our study aligns with workplace learning approaches, paying particular attention to both beliefs (conceptualizations) and actions (how they describe their actual lifelong learning practices).

### Participants and Setting

We sampled from physicians on faculty (academic physicians) at the University of California, San Francisco. We intentionally selected academic physicians who interacted with trainees since teaching opportunities can be an important part of a physician’s choice to work in an academic environment. We sent email invitations in batches of 30 to eligible academic physicians and sought to maximize variation based on specialty, rank (assistant, associate, or full professor) and health system (community-based, county/public, university, Veterans Affairs). This process allowed us to monitor the diversity of our sample and adjust recruitment to include all ranks, systems, and multiple specialties. We ceased data collection after 34 interviews, a decision based on sample diversity (Table 1) and analytic sufficiency [29]. Analytic sufficiency was based on patterns and richness of evidence across the dataset; we did not sample with the intent to compare patterns among specialty, rank, or health system.

**Table 1:** Descriptive information about Faculty Participants (n = 34).


CHARACTERISTICS		N

**Gender**	Woman	20

	Man	14

**Health System**	University	21

	Community-Based	5

	County/Public	4

	Veteran’s Health Administration (VHA)	4

**Specialty**	Internal Medicine	10

	Pediatrics	6

	Anesthesia	5

	Psychiatry	3

	Surgery	3

	Emergency Medicine	2

	OB-GYN	2

	Family Medicine	1

	Neurology	1

	Pathology	1

**Faculty Rank**	Assistant Professor	15

	Associate Professor	8

	Full Professor	11

**Years of experience as an educator**	Median	8 years

	Range	1–48 years


### Data Collection

We developed semi-structured interview questions based on literature on lifelong learning and physician competency domains [4,30]. Questions asked participants to explain what lifelong learning means to them, what areas they focus on, and how they pursue lifelong learning in these areas. If participants did not mention lifelong learning in domains other than medical knowledge and clinical/procedural skills, we asked for their thoughts on lifelong learning in areas such as interpersonal relationships, professionalism, and emotions (Appendix 1).

One author (SC) conducted all interviews from June to December 2019 via Zoom with participants’ consent. All interviews were recorded and transcribed through a professional transcription service. Interviews lasted 39–87 minutes. Each transcript was checked for accuracy and de-identified by one author (SC). Our study was approved by the UCSF institutional Review Board and deemed exempt (IRB# 19-27900).

### Data Analysis

Data analysis began after the first interview and continued throughout data collection [31]. We began by coding interviews using low inference categorical codes aligned with our interview questions. Then, consistent with the familiarization phase of thematic analysis [31], we summarized excerpts describing participants’ conceptualizations and actual processes of lifelong learning. At this point in our analysis, we revisited the literature on lifelong learning and, upon reading Biesta’s work in adult education [24], recognized a connection between our interpretation of the purposes present in participants’ descriptions and the purposes Biesta articulated. We organized our findings according to three purposes of lifelong learning that reflect a combination of our own interpretations and Biesta’s writing (maintaining competence, fulfillment and growth, professional stewardship). Following this initial coding, SC and BO’B further reviewed coded excerpts and engaged in regular discussions to construct themes that captured participants’ conceptualizations of lifelong learning, including purposes, content areas (e.g., medical knowledge, clinical/procedural skills, communication, emotional awareness), processes, and to determine when we reached analytic sufficiency [29]. Analysis for our second research question followed a similar process beginning with familiarization that led to development of codes that captured how participants engaged in lifelong learning (contextual, social, individual processes) and identification of themes related to these processes.

## Results

Our findings are based on interviews with 34 academic physicians from ten specialties (20 women; 15 assistant professors, 8 associate professors, 11 full professors). (Table 1) We organized our findings according to our research questions. First, we present physicians’ conceptualizations of lifelong learning, which we inferred from participants’ descriptions and examples of lifelong learning. These conceptualizations are organized around three overarching purposes of lifelong learning (maintaining competence, providing personal growth and fulfillment, and engaging in professional stewardship), which offer insights into the breadth of content and processes physicians associate with lifelong learning. We then present findings related to our second research question about how physicians actually engage in lifelong learning, with themes showing the multilayered nature of lifelong learning practices, challenges faced, and variation by content and purpose.

### Physicians’ Conceptualizations Of Lifelong Learning

#### Lifelong Learning to Maintain Competence: *“Constantly updating my knowledge and concepts in an iterative fashion”*

Participants primarily described lifelong learning in ways we interpreted as serving the purpose of maintaining competence as a physician. They emphasized the importance of “keeping up-to-date” with the literature, methods, guidelines, and research to support clinical decision-making, noting that, in a rapidly evolving field, “anyone who isn’t involved in lifelong learning as a physician is hopelessly out of date very quickly”(P124). As such, participants viewed lifelong learning as a “necessary process” for clinical excellence throughout one’s career and “an integral part of what we do every day”(P109), driven by the fundamental purpose to “provide patients with the best care”(P134) and to provide trainees with the best possible education. Yet, a common theme in this conceptualization was a sense of never doing enough, finding it “almost impossible to stay up to date”(P105), and feeling like their process should be more “systematic.”

The focus on maintaining competence skewed the content toward medical knowledge and clinical or procedural skills, though many participants also identified areas such as communication, patient relationships, teamwork, leadership, emotions, quality improvement, and DEI (diversity, equity, inclusion) as areas they considered important for maintaining competence. One participant explained that much like medical knowledge, “the efficacy of my communication with patients or with trainees and how I continue to integrate feedback from a given clinical interaction into the way I approach my next difficult conversation with a patient” is important because “over time the themes of difficult conversations may evolve and so being able to adapt my communication to those things” contributes to physicians’ competence. Participants found lifelong learning in these areas more challenging than medical knowledge and clinical skills because “a lot is experiential, [and I’m] not sure what the best strategies are” (P108), many of these topics were not covered in their training so they did not develop learning strategies, and they recognized that some of the learning required doing things that are difficult- “it takes an openness to look at your own biases”(P116). By contrast, a few participants viewed these as skills that people needed to learn but did not require ongoing learning to maintain competence as a physician.

#### Lifelong Learning as a Source of Personal Growth and Fulfillment: *“People who engage in lifelong learning enjoy life and work much more”*

Though less common, we found that participants also associated lifelong learning with a sense of personal fulfillment that came from an alignment of lifelong learning with a core aspect of their character or identity. Participants saw engagement in lifelong learning as “part of who I am as a physician”(P108), as someone who is “always curious and always trying to think about medicine…best practices…your own practice…every case.” (P122) Some expressed a love for learning, “I’m curious, so I really enjoy and get so much personally out of that. I really love aligning my work with the ability to still be a learner.” (P125)

Lifelong learning invigorated participants and kept them thriving. One participant explained the importance of lifelong learning for “maintaining the joy in your practice; maintaining that curiosity and spark and novelty of the work. Otherwise, I think you just sort of get stagnant.” (P111) Thus, participants viewed lifelong learning as “not a chore”(P125), but as a source of fulfillment through learning, finding it “easy to engage in that work, because it’s…for the betterment of the people I’m going to be teaching or for the patients I’m going to be caring for, the folks I’m going to be mentoring.” (P111)

Learning for this purpose could involve any content area, including medical knowledge and clinical skills as well as communication, medical education, quality improvement, and leadership. The key distinction between lifelong learning for personal growth/fulfilment and for maintaining competence was the opportunity to delve deeply into a topic, often for reasons driven more by curiosity or interest rather than immediate need to know or improve. This learning could involve formal trainings, as articulated in “I found that going back to do that [relationship centered communication] on the advanced level has been very rewarding, including the formal CME courses as well as trying to teach it” (P125) and/or informal learning through “self-study” or conversing with or observing colleagues, for example “I’m a very social learner when it comes to learning about the more humanistic sides of medicine. So I really enjoy shadowing. And I found a lot of value in that, (P134).” Often this learning occurred on participants’ own time rather than as part of their workday, as illustrated in comments such as, ”I also want to improve things like organizational skills. So I’ve been reading things in my own personal time, like skills to be an effective person.” (P131)

#### Lifelong Learning as Professional Stewardship: *“I think lifelong learning by example is one of the strongest things that we as physicians can do for our learners”*

A third conceptualization of lifelong learning reflected participants’ commitment to sharing their learning with trainees and colleagues for the greater good of the profession and patient care. They consciously modeled and discussed their own learning with trainees, recognizing that, “A really important part of our job is to be learning and growing all the time… it’s also very important for me to instill this in the learners that I encounter.” (P108) Participants sent trainees articles they had read, and modeled “communication skills…it’s definitely something that I want to model…that I continue to work on improving.” (P120)

Participants described a sense of responsibility to share their learning with colleagues, recognizing the mutual benefit of learning from each other: “I consider myself expert and focused on…a few particular areas of my specialty, but I also consider it my job to keep others informed about newer evidence in those areas. And I rely on other faculty to do similar for areas where I do not focus as much.” (P109) Although correcting a colleague’s mistake may support others’ lifelong learning, only one participant discussed this and explained, “it’s not the culture” among physicians to provide direct feedback on a decision that is contra-indicated. Instead, they might provide indirect feedback via a note in the medical record, “hoping that they read that and incorporate that in some way.” (P134)

### How Academic Physicians Engage in Lifelong Learning

Participants described a combination of formal and informal practices to support their learning that included systematic, deliberate approaches and in the moment “reactive” approaches based on the “need to know” that are not “integrated into a larger framework”(P106) for later retrieval or lasting improvement. Many found it relatively easy to describe their lifelong learning practices to support clinical medicine and felt confident about their approach. They had more difficulty describing, and felt less confident about, their practices in domains with interpersonal, relational, or emotional components.

*“Clinically, I feel quite good about my approaches to lifelong learning…I have an effective way of staying on top of things and can really be the kind of the clinician that I want to be – And then I think some of the more advanced aspects, whether it’s emotional awareness or relationship-centered communication … is something that I’m always developing. … the hard part for me is how do you actualize it in the real world?”(P125)*

Participants also described an attitudinal component to their lifelong learning, emphasizing the importance of “not get[ting] complacent with the body of knowledge that you have.” (P118) They explained how maintaining curiosity and openness to new information, new approaches, and new experiences established a “mindset [that] forms the foundation of how I believe we can position ourselves to be open to evolution as clinicians and to create a fertile environment for us to continue to learn.” (P121) Qualities such as the capacity to be “intellectually honest,” willing to say “I don’t actually know” (P127), and “humble about my knowledge base and being aware of my limits…never actually feeling like a master”(P101) appeared in many participants’ discussions of lifelong learning.

Participants described many specific lifelong learning practices, which we categorized as *contextual*; *social*; and *independent processes*. We briefly describe each below along with challenges mentioned in each category. Supporting quotes appear in Table 2.

**Table 2:** How Physician-Educators Engage in Lifelong Learning: Contextual, Social, and Independent Processes.


ENGAGEMENT IN LIFELONG LEARNING	SUPPORTING EVIDENCE

Contextual (work environment and systems/structures supporting lifelong learning)	*Accountability* “One of the aspects I love most about working in academic medicine and having students is just how honest and current everyone keeps you to be cutting edge.” (P125) “My residents are getting smarter than me. Honestly they are my main motivator…I need to know I offer something to my residents.” (P129) *Access to research* “being at an academic center, I’m lucky enough to be in a place where just being there I’m in the milieu of learning about new research and what the latest best practices are for different things… Even if I’m not actively doing the research, I am privy to it and learning about it because I’m involved in it through my clinical work.”(P107) *Access to training, faculty development* “We do faculty development within our own division. Another benefit of being at a large academic center is there’s a lot of ongoing faculty development that you literally have to just be present and it comes to you.”(P107) Observing and emulating others

Social (learning through interaction with others)	*Observing and emulating others* “I shadow my own colleagues and see how they teach, especially the ones who might be more up-to-date clinically in certain areas than I am.” (P103) “I pick up things by assisting other surgeons and watching what other people do in surgeries.”(P116). *Talking with colleagues* “talking to my colleagues is a huge way that I learn how somebody would manage somebody else differently. It’s probably the most common day to day thing that I use to learn about managing certain conditions” (P102) *Learning with and from trainees* “I learn a lot every time I teach, actually, because the students coming in know totally different things than what I learned, what I knew when I came in as a medical student, and have different life experiences.”(P108) *Seeking and processing feedback from trainees, patients, colleagues* “I think I’m pretty good about asking learners very deliberately for constructive feedback at the end of my time with them. And if I get feedback on how interpersonally something was, or professionalism wise, or questions about it, that might prompt me to self-improve. So, seeking feedback is a way that I learn.” (P130) “there are points in time where you get feedback. And you can get it from patients… I think medical translators are actually a goldmine for communication…You can see it in people’s eyes, right? They just glaze over, they don’t know what you’re talking about.” (P113) *Building networks of mentors and trusted colleagues* “I recognize when there are people that I really want to emulate, and I tried to bring them in and make them mentors of mine. And I’ve done that with a couple of people.” (P134) “I’ve reached out to senior faculty who I admire or have a lot of trust in their skills, whether it is as a leader or a clinician or an educator and have asked them to be somebody that I can call upon when I need help. I have a few people that I go to regularly.” (P107)

Independent (learning on own)	*Looking things up* “there’s always questions that arise just from the individual encounters, that I will look up the answer to or try to refresh my memory on. So, I’ll turn to medical resources to try and learn more, or answer a specific question that was brought up by that individual.” (P101) *Reviewing resources* “most of it is honestly on my own time, meaning whether I have signed up for a bunch of listserv and various emails …And so those emails tend to be quick blurbs about certain topics, and so I get two are three of those a week.”(P120) *Pursuing further training* “if there are lectures on campus, or when I go to national conferences, I’ll both go to sessions that are things that I’m interested in and then go to sessions on things that I know are gaps for me. And then try to develop the skillset.” (P111) *Reflection and Introspection* “A lot of introspection … some days you feel good about how you handle tough situations and you give yourself a pat on the back. And some days you go home and say, ‘Oh my God, I bombed with that one.’ And you try and learn from it…and say, ‘Hey, I was very worked up in the situation and these are the circumstances surrounding this interaction that I had with this person. And, yes, they were wrong, but that doesn’t make it okay for me to be wrong also. And these are the things that I can do from here on out.” (P118)


**Contextual:** Working in an academic medical center made it “easy” for participants to engage in lifelong learning through abundant access to professional development and other learning resources/supports; discussions with trainees and colleagues; and exposure to cutting-edge research. A sense of accountability drove participants’ lifelong learning to maintain legitimacy among peers, combat “imposter syndrome,” or establish credibility among trainees who may expect faculty “to defend the things that you’re saying.” (P111) Participants valued working in an environment that normalized admitting one’s limitations and asking for help, but also realized the limits of a culture that relies on self-awareness and self-assessment to guide learning. Several, particularly those early in their career, mentioned a sense of isolation and the challenge of entering practice and no longer having feedback to identify areas for improvement since “there’s nobody actually observing you and telling you whether you’re doing a good job.” (P134) The desire for better systems to identify gaps was clear, as one participant explained, “talk to anybody and they all believe that they’re keeping up, but studies show we’re not. … So how do we do lifelong learning where it’s actually hitting people’s deficiencies?” (P132)

**Social:** Without formal structures for feedback, participants relied on social learning processes such as observing others, conversing with colleagues and trainees, asking for feedback, and connecting with trusted mentors. Observation when working with colleagues could tap into colleagues’ knowledge and reasoning processes, or offer ideas for procedural techniques, but more often yielded learning about interpersonal and communication skills. One participant described the value of observing trainees to “identify things that I want to emulate… to sort of borrow things and that’s how I grow interpersonally”(P106). Discussions in real time also presented learning opportunities, such as during bi-directional teaching with trainees who “often have so much more up-to-date knowledge than the faculty.” (P106) Discussions also occurred in regular forums where colleagues “shared their internal world” as they worked through psychologically or emotionally challenging encounters. Several participants valued debriefing with a trusted mentor for feedback and “guidance on what I need to be working on and how I need to be working on it.” (P107)

**Independent:** Participants’ independent lifelong learning generally occurred ‘just-in-time’ (looking things up as needed); through systematic, intentional activities (attending conferences, workshops; reviewing resources to stay up-to-date; meeting recertification requirements; or pursuing further training); and through reflection (exploring their emotional responses, interactions or decisions for improvement). Much lifelong learning in the interpersonal and emotional domains occurred through further training or through reflection on experience, but participants considered these areas “difficult … to have good self-awareness around” (P119) and as “more personal” and thus more sensitive. Participants suggested that, compared to lifelong learning of clinical content, there were fewer resources and less normalized or ingrained processes or practices for lifelong learning in the interpersonal and professionalism domain.

## Discussion

Our findings suggest that maintaining competence reigns as the primary way academic physicians conceptualize the purpose of lifelong learning, though we also found ample evidence of two additional purposes – supporting professional growth and fulfillment and professional stewardship. While the responsibility to keep up-to-date for patient care (competence) could, in theory, include many content areas, much of this learning was driven by the narrow scope of medical knowledge or skills needed at the point of care. This learning-in-the moment relied on processes that some felt were insufficient and less effective than they desired. By contrast, lifelong learning for the purposes of professional growth and stewardship were generally driven by curiosity, interest, and interactions with trainees or colleagues – all of which required a deeper level of engagement with broader content and processes that tended to feel more satisfying and rewarding. We contend that all three purposes are important for academic physicians and the profession to thrive, though this ideal may be increasingly difficult to achieve in academic medical environments. Our findings also show that participants’ conceptualizations of lifelong learning spanned a broad range of content areas and learning processes, though participants found it easier to articulate their lifelong learning processes for medical knowledge and clinical or procedural skills than for other domains (e.g., communication, emotions, interpersonal skills). Our discussion considers the value of conceptualizing lifelong learning for purposes beyond maintaining competence and opportunities to better support lifelong learning in multiple domains of medical practice.

Lifelong learning is necessary to maintain competence, but the value of lifelong learning extends beyond this purpose. Participants described additional ways they valued lifelong learning, even if not directly improving their ability to deliver quality patient care. Consistent with the concept of person-job fit [32], participants described a sense of joy and fulfillment associated with alignment between personal characteristics they value (e.g., curiosity, inquisitiveness, skepticism) and job characteristics that attracted them to medicine (e.g., the opportunity for ongoing learning about scientific advancements and human experiences). The literature on person-job fit suggests that the stronger this alignment, the more likely employees are to find their work meaningful and satisfying [32,33]. As such, the value of lifelong learning for personal growth and fulfillment may warrant further attention and support, particularly if it can serve to protect against burnout [34,35].

The conceptualization of lifelong learning as a form of stewardship, or commitment to supporting and instilling lifelong learning in others, shows how strongly physicians internalize lifelong learning as part of their identity as physicians. Participants viewed it as their responsibility to cultivate this dimension of professional identity in trainees by modeling lifelong learning or co-learning with trainees [36]. This responsibility extends beyond trainees to support colleague’s lifelong learning by sharing expertise and desiring feedback from colleagues, although participants noted this can be challenging. Other studies have found a general reluctance among physicians to provide feedback to colleagues about outcomes from their clinical decisions, largely because they worry the feedback might be unwelcome or cause negative emotions that harm relationships [37,38]. While physicians generally recognize the shared responsibility and mutual benefits of engaging in lifelong learning, professional norms and feedback systems may need shifting to realize the full potential of feedback among colleagues to enhance lifelong learning.

The focus on lifelong learning to maintain competence may help explain why medical knowledge and clinical/procedural skills received far more attention than interpersonal, emotional, and professionalism aspects of medical practice. Relational and emotional aspects of practice such as compassion, grief, distress, and well-being are areas for ongoing learning and growth, yet many clinical practice environments do not optimally support or prioritize such learning [35,39,40]. Furthermore, these areas are difficult to teach and define with measurable outcomes, which likely contributes to fewer training opportunities and less representation in maintenance of certification processes compared to clinical knowledge and skills.

Unsurprisingly, participants had difficulty articulating their lifelong learning in broader areas regarding relationships, emotions, and well-being, though they recognized them as important. The lack of awareness, intentionality, and support for learning in these areas likely has implications for trainees – if their supervisors are not openly discussing and modeling their efforts to improve relationships, emotions, and well-being, trainees are less likely to do so themselves and a professional culture resistant to discussing vulnerability persists [35,41,42]. The growth of resources and literature on CPD in areas such as relationship-centered communication [43], well-being [44,45], anti-racism [46], and compassion [47] along with the inclusion of such topics in medical school and residency curricula are promising signs that learning in these areas is increasingly accepted as necessary for excellent patient care. However, cultural shifts also require investment in structures and systems that reinforce the contextual and social processes by which lifelong learning occurs, particularly when demanding clinical environments and productivity pressures increasingly limit capacity for independent lifelong learning.

Although much of the literature on lifelong learning focuses on formal, structured educational opportunities, most of what participants in our study described was informal and relatively unstructured. Informal learning can be problematic as it often depends on physicians to self-identify gaps, which is unreliable [48], places high burden on physicians to find the best sources and methods for learning [16], and can cause stress as there is so much more to learn than can be learned. Yet, informal learning has beneficial features including relevance to practice, timeliness, customizability, and alignment with personal needs or interests. Recent literature on CPD recognizes the importance of both embracing informal workplace learning opportunities and improving formal learning experiences by making them more outcomes-oriented and using pedagogical principles informed by the learning sciences [49,50]. Creating the optimal environment for lifelong learning likely involves contextual changes such as restructuring clinical work to create more time and space for social learning processes such as observation, feedback conversations, co-learning, and participation in high-quality formal learning CPD activities such as case-based conferences, interactive workshops, and journal clubs. Additionally, recent CPD literature encourages improvements in performance data as a source of feedback to guide self-regulated learning [16]. While participants in our study expressed desire for more feedback on their performance as clinicians and educators, few described reliable receipt or use of such information.

### Limitations

Our study occurred in an academic setting, which many participants recognized as important in their ability to engage in lifelong learning opportunities. Physicians in community-based or corporate settings may have different ways of engaging in lifelong learning and may find it more difficult to integrate lifelong learning into daily practice. Our findings are based on perceptions collected through interviews, which provides only a partial understanding of how lifelong learning occurs in clinical practice. Observational data could provide a more complete picture of at least the visible aspects of lifelong learning. Interviews occurred with academic physicians from a single institution prior to the COVID-19 pandemic, which gives us insight into the norms and culture of this institution at a particular point in time. Conducting similar studies at other institutions and post-pandemic would help to clarify the conceptual generalizability of our findings.

## Conclusion

Academic physicians in our study described the need to maintain competence for patient care as their primary purpose for lifelong learning, but also indicated a strong motivation based on the joy and fulfilment they gained from lifelong learning and their sense of responsibility to teach others. These latter purposes yield important benefits for sustainability and well-being of physicians that extend beyond direct patient care. We also found medical knowledge and clinical/procedural skills as the primary foci of attention across all three purposes. Though participants acknowledged the importance of learning in broader domains of professional practice, fewer available opportunities and difficulty articulating learning processes suggests these areas are less frequently conceptualized as areas for lifelong learning, less well supported, and harder to prioritize. A comprehensive approach to lifelong learning will require embracing learning as a more contextual, social, and purpose-driven process that combines outcomes-oriented formal learning opportunities with informal, interactive workplace-based learning that encompasses a broader range of content.

## Appendix 1: Relevant Questions from Interview Guide

1. When you think about professional lifelong learning – what does that mean to you?
  - What are some examples of topics or areas in which you pursue lifelong learning? (IF they ask, any area is fine, not just clinical)
  - How does this lifelong learning occur for you? (OR How do you go about lifelong learning in these areas?)
  - What cues you to pursue learning (e.g., how do you identify or know when you need to learn something?)
  - To what extent is this learning visible to learners?
  - IF ONLY MENTION CLINICAL IN INITIAL RESPONSE – Most of what you mentioned pertains to clinical knowledge and/or skills/procedures. I am curious about other domains such as communication and interpersonal skills, professionalism and emotional awareness. To what extent would you consider these areas of lifelong learning?
    - How do you engage in lifelong learning in these areas?
    - How competent do you feel about your lifelong learning in these areas (particularly compared to the areas you mentioned initially)?
    - To what extent is this learning visible to learners?
2. Are there ways, in addition to what we discussed above, that you teach or model lifelong learning for learners in clinical settings?
